# Chronological changes in rhinitis symptoms present in school-aged children with allergic sensitization

**DOI:** 10.1371/journal.pone.0210840

**Published:** 2019-01-17

**Authors:** Michelle J. Suh, Jin A. Park, Suk Won Chang, Jeong Hong Kim, Keun-Hwa Lee, Seong-Chul Hong, Hye-Sook Lee, Ju Wan Kang

**Affiliations:** 1 Department of Otorhinolaryngology, Jeju National University School of Medicine, Jeju, South Korea; 2 The Environmental Health Center (Atopic Dermatitis and Allergic Rhinitis), Jeju National University, Jeju, South Korea; Istituto di Fisiologia Clinica Consiglio Nazionale delle Ricerche, ITALY

## Abstract

**Introduction:**

It is difficult to accurately predict the natural course of allergic rhinitis (AR), because it is affected by a wide variety of environmental influences, as well as genetic predisposition. Considering the high prevalence of allergic rhinitis in children and adolescents, caregivers should be given appropriate information regarding the disease course. This study aimed to understand the prognosis of allergic rhinitis by examining the relationship between allergic sensitization and rhinitis symptoms during this developmental period.

**Methods:**

This cross-sectional study included 1069 children aged 9–16 years from the Korean International Study of Asthma and Allergies in Childhood Survey database who had completed health questionnaires, and for whom skin prick test results were available. Data were collected during May 2016. The distribution of sensitization and allergic symptoms was compared by age groups (elementary, middle, and high school). Data were analyzed using linear-by-linear analysis.

**Results:**

Sensitization to at least one tested allergen differed by age (59.2%, 58.3%, 68.2%, in elementary, middle, and high school students, respectively; p = 0.025), and seasonal allergen sensitization (35.0%, 37.1%, 53.9%, respectively) increased with age (p < 0.001). Conversely, the proportion of rhinitis symptoms among sensitized children decreased as age increased (58.80%, 52.90%, 49.70%, respectively; p = 0.047). However, the rate of non-allergic rhinitis was age-independent.

**Conclusion:**

With increasing age during childhood and adolescence, symptomatic allergic rhinitis decreases; thus, subclinical allergic rhinitis increases. This suggests that the symptoms of later-sensitized children are less clearly manifested, or that the symptoms reduce as previously sensitized children mature. This should be clarified further in a longitudinal study.

## Introduction

The worldwide prevalence of allergic rhinitis (AR) differs widely across countries and regions because the causative allergens and aggravating factors depend on the living environment. The prevalence of AR shows an increasing trend worldwide, especially among school-aged children [1,2]. Active allergic disease reduces children’s quality of life [3,4], and imposes a considerable socioeconomic burden [4,5]. Therefore, it is of interest to determine whether AR in a specific child will deteriorate or develop into asthma or other allergic diseases; moreover, caregivers require counseling regarding how their children’s diseases are likely to change over time. It is presumed that the prevalence of AR increases from childhood to young adulthood, and decreases thereafter [6,7]. Nevertheless, there has not been sufficient research regarding the characteristics of AR in school-aged children.

Recent studies based on a more controlled environmental background have shown a decline in the prevalence of AR as children mature (34.5% in preschool-aged children vs. 16.2% in adults) [8–11]. However, previous studies only showed changes in prevalence with aging, and there have been no studies regarding how existing allergic symptoms have changed with age in sensitized children.

This study investigated the clinical characteristics of AR in Korean school-aged children by analyzing the relationship between age-specific allergen sensitization and the presence of rhinitis symptoms; the goal of the study was to provide counseling guidelines to parents and caregivers regarding changes in children’s AR characteristics over time.

## Materials and methods

### Study population

Six elementary schools, three middle schools, and four high schools located in Jeju and Seogwipo City, Jeju Island, were selected. Children (students) from third to fifth grades in the elementary schools, were invited to enroll in this study, as were students from the first and second grades in the middle and high schools. A detailed description of the study was provided to children and caregivers, and written informed consent was obtained from parents. This study was approved by the institutional review board of the Jeju National Hospital University, in accordance with the Declaration of Helsinki. Data were collected in a manner that maintained the confidentiality of the children’s records.

**Fig 1:**
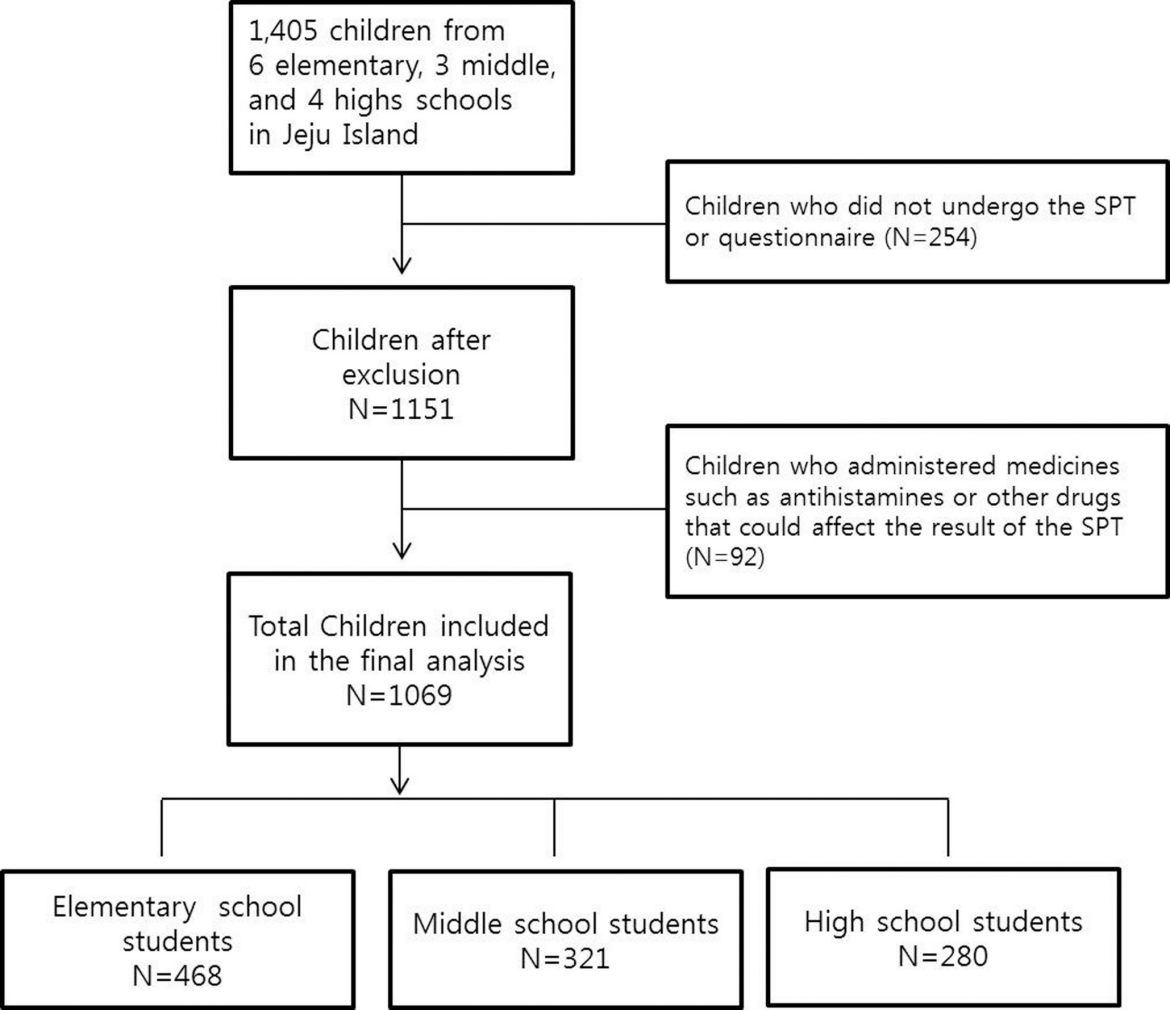
Flow chart of children included for analysis.

Children and parents were asked to fill out a questionnaire. Appropriately trained nurses performed skin prick tests (SPTs) in May 2016. Children who either did not respond to the questionnaire or did not undergo the SPT were excluded. Furthermore, children who used drugs that might affect the results within 7 days before the test, such as antihistamines, were excluded. Finally, 141 elementary, 108 middle school, and 97 high school students were excluded, and 1069 children (468, 321, 280, respectively) were included in the analysis (Fig 1).

### Assessment

Sex, school grade, and presence of rhinitis symptoms were ascertained from the self-reported Korean International Study of Asthma and Allergies in Childhood (ISAAC) questionnaire. In the questionnaire, the presence of rhinitis symptoms was judged by the following question: “Has your child ever had a problem with sneezing, a runny nose, or a blocked nose when he/she did not have a cold or the flu?” (S1 Table)

An allergic skin prick test using 26 common aeroallergen extracts was performed including *Dermatophagoides pteronyssinus*, *Dermatophagoides farinae*, cat, citrus red mite, dog, *Cladosporium*, *Alternaria*, Timothy grass, mugwort, barley, rapeseed, Japanese hop, Japanese cedar, rye grass, hen’s egg (white), tuna, cow’s milk, soy flour, mussel, yolk, shrimp, chicken meat, pork, peach, peanut, and wheat flour. Japanese cedar allergen was purchased from Greer Laboratories Inc. (Lenoir, NC, USA) and citrus red mite allergen was prepared as described in a previous study [12]. All other allergens were purchased from Allergopharma (Reinbek, Germany).

Histamine hydrochloride, 1 mg/ml (Allergopharma) was used as positive control and a normal saline solution with 50% glycerin was used as a negative control. Trained researchers introduced allergens into the epidermis by using a 23-gauge lancet. The results were recorded after 15 minutes by measuring the size of each wheal as the mean of (A) the longest diameter and (B) the diameter perpendicular to the first axis at the midpoint (i.e., (A + B)/2). A positive SPT was defined as an allergen that elicited a wheal of ≥ 3 mm. Allergic sensitization was established and AR was diagnosed based on a positive response to more than one proven allergen by SPT. When a child had at least one positive SPT result and was positive for “current symptoms and signs for allergic rhinitis” on the Korean ISAAC questionnaire, AR was diagnosed.

### Statistical analyses

Categorical variables are expressed as percentages. The proportion of sensitization or presence of symptoms was compared between age groups using a linear-by-linear association analysis. PASW Statistics 17 (SPSS Inc., Chicago, IL, USA) was used for all analyses. A p-value of < 0.05 (two-tailed) was considered significant.

## Results

### Population characteristics

In total, 1069 children, all of whom underwent SPTs and completed the ISAAC questionnaire, were included in this study. The group consisted of 554 (51.8%) females and 515 (48.2%) males; the mean age was 12.5 years (standard deviation: 2.6 years, range 9–16 years). The included children consisted of 468 (43.8%) elementary, 321 (30%) middle, and 280 (26.2%) high school students (Table 1).

**Table 1:** Characteristics and demographics of children in this analysis.

Feature	Study population
Total number	1069
Sex (Male:Female)	515 (48.2%): 554 (51.8%)
Age (years)	12.5 ± 2.6 [9–16]
Age Groups	
Elementary school	468 (43.8%)
Middle school	321 (30%)
High school	280 (26.2%)

Mean and standard deviation are reported for age. Numbers in brackets reflect the age range of included children.

### Prevalence of allergic sensitization

**Fig 2:**
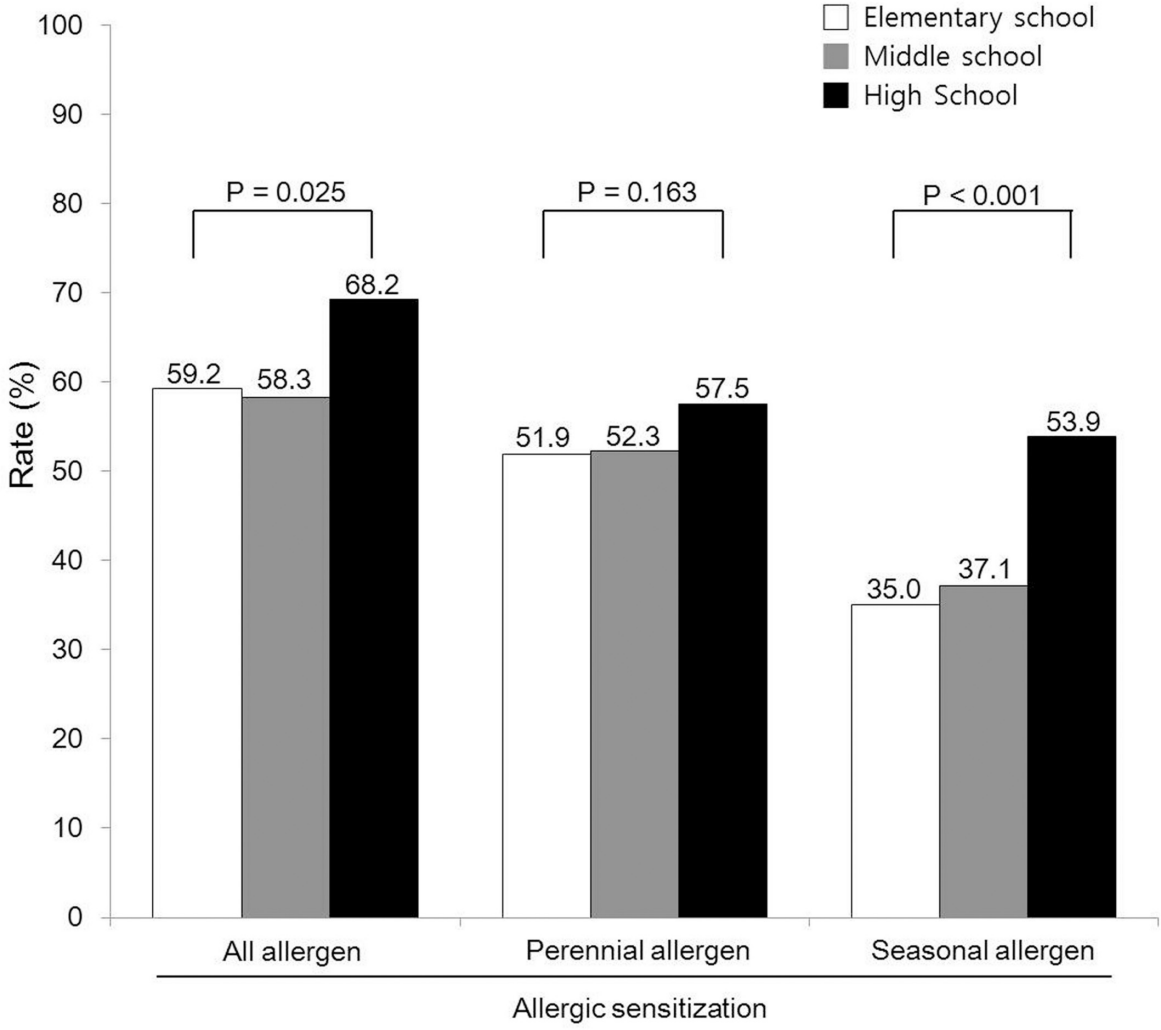
Allergic sensitization according to age group. *P*-values were calculated using linear-by-linear association analysis. Statistical significance was defined as p < 0.05.

The prevalence rates of sensitization to at least one of the tested allergens were 59.2% (277/368) for elementary, 58.3% (187/321) for middle, and 68.2% (191/280) for high school students; these rates were significantly different (p = 0.025). When we categorized allergens into perennial and seasonal allergens, there was no difference in the perennial allergen sensitization rate among the age groups (51.9%, 52.3%, and 57.5%, respectively; p = 0.163); however, seasonal allergen sensitization increased with age (35.0%, 37.1%, and 53.9%, respectively; p < 0.001) (Fig 2).

### Symptom presence according to age and allergic sensitization

**Fig 3:**
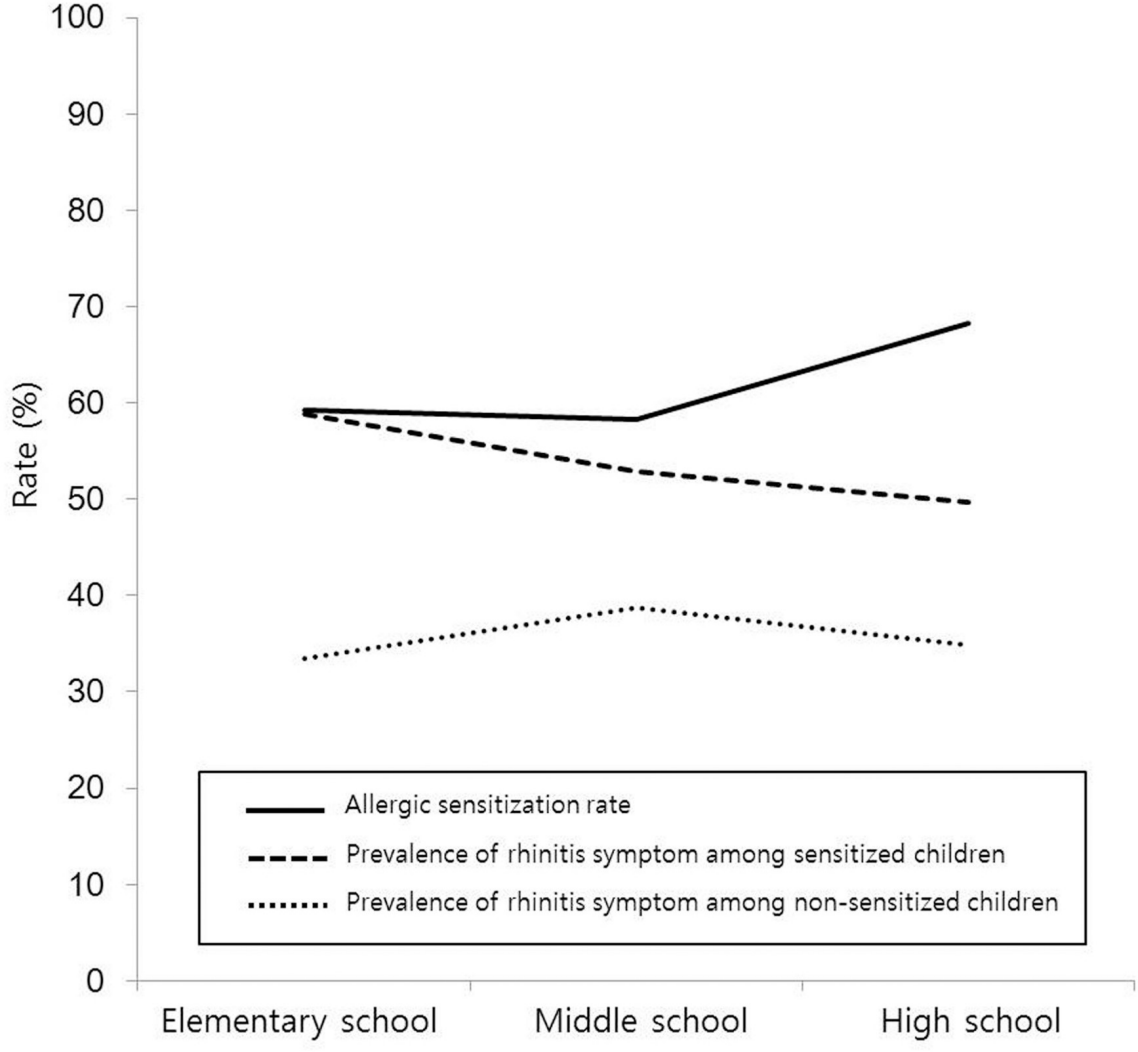
Prevalence of rhinitis symptom in sensitized and non-sensitized children according to age group.

The overall and age-specific prevalence of AR was 33.4%; it was 34.8% in elementary school, 30.8% in middle school, and 33.9% in high school students. A total of 163 (58.80%) of 277 sensitized elementary school students, 99 (52.9%) of 187 sensitized middle school students, and 95 (49.7%) of 191 sensitized high school students were symptomatic; thus, there was a tendency for symptoms to decrease with age (p = 0.047) (Fig 2). However, the prevalence of rhinitis symptoms among unsensitized children (elementary school: 40.8% [191/468], middle school: 41.7% [134/321], and high school: 31.8% [89/280]) did not significantly differ (33.5%, 38.8%, and 34.8%, respectively; p = 0.674) (Table 2 and Fig 3).

**Table 2:** Prevalence of rhinitis symptoms in sensitized and non-sensitized children according to age group.

		Sensitized		Non-sensitized	
	N	All	Rhinitis symptom (+)	All	Rhinitis symptom (±)
Elementary	468	277 (59.2%)*	163 (58.8%)*	191(40.8%)*	64 (33.5%)*
Middle	321	187 (58.3%)*	99 (52.9%)*	134 (41,7%)*	52 (38.8%)*
High	280	191 (68.2%)*	95 (49.7%)*	89 (31.8%)*	31 (34.8%)*
P value		0.025†	0.047†	0.025†	0.674

†Significant differences (p < 0.05) between groups in a linear-by-linear association analysis.

*Data are presented as number (rate)

## Discussion

This study investigated the relationship between allergen sensitization and symptomatic rhinitis in children of different age groups to gain an understanding of the course of AR. Surprisingly, although the rate of allergen sensitization increased with age, the extent of rhinitis symptoms in sensitized children decreased with age; in contrast, non-allergic rhinitis symptoms revealed a growth-independent pattern.

Previous studies have investigated allergen sensitization and overt allergic symptoms. Several studies have shown that sensitization to inhaled allergens increases with age in children [13,14]. However, in studies of adults, the frequency and severity of AR tended to decline with age [15,16]. One of the factors affecting sensitization to various allergens is the living environment, which is related to the frequency of exposure [17]. The sensitization rate is speculated to incrementally increase with frequent exposure to the antigen. Compared with children, adults are more frequently sensitized to nearly all pollen allergens, which are representative of seasonal allergens. As children mature, the likelihood of exposure to outdoor antigens increases; this was supported by the current findings indicative of increasing seasonal allergen sensitization with age. Subsequent allergen exposure in pre-sensitized children leads to an increase in basophils and mast cells, which mediate IgE release; this results in symptoms such as rhinorrhea, nasal obstruction, or itching sensation. However, a previous study found that sensitization and overt symptoms are not always correlated; thus, the etiology has not yet been fully explained [6].

Sherrill et al. reported that early-sensitized children had a high risk of asthma occurrence and severe allergic symptoms, whereas mild symptoms were present in late-sensitized children [18]. Although the present study is cross-sectional and we had no information regarding the time point when each of the children became sensitized, the aforementioned research and other recent results suggest that the cumulative prevalence of sensitization increases with age, and that less obvious symptoms are present in newly-sensitized individuals.

A previous study showed that overall AR symptoms tend to become milder with age, and that allergic reactivity decreases after an average follow-up of 22.9 years, with no direct association between changes in symptom severity and test results [19]. The incidence and severity of allergic symptoms both tend to decrease with age. The decline in the onset of allergic symptoms observed with ageing might be due to reductions in serum total and specific IgE levels [20]. Our results may also be a sign of this long-term change.

There is another possible explanation for age-related changes in the level of causative allergens; the threshold may be lower at a younger age. When exposure to an allergen is below the threshold needed to elicit symptoms, weak inflammatory infiltration occurs in the nasal mucosa; this leads to absence of symptoms [21]. Notably, low-grade, continuous, subclinical allergic inflammation has been reported, even in the absence of symptoms, in individuals with seasonal AR and those with perennial AR [22]. Although there is no evidence supporting this suggestion that the threshold for allergen exposure changes with age, the increase in the proportion of children with subclinical AR in the present study suggests that this may be a possibility. Future studies should investigate whether subclinical AR persists silently, even into adulthood.

As a management strategy for allergic diseases, the primary step is prevention of immune sensitization; the second is prevention of allergic symptom development in sensitized individuals; and the third is treatment of allergic diseases [23]. The findings of the present study, which was designed to elucidate changes in the process between the first and second stages described above, may form the basis for identifying the pathophysiology underlying the transition from sensitization to manifestation of allergic symptoms.

This study had several limitations. First, we showed that asymptomatic allergic sensitization increased with age in school-aged children; however, asymptomatic skin sensitization may be a risk factor for later allergy development [24]. Therefore, longitudinal studies in children with asymptomatic sensitization are needed to confirm the clinical implications of our findings. Second, SPTs were used to confirm allergic sensitization; however, SPT results could change over time and may also be false-positive or false-negative. Therefore, these results should be cautiously interpreted. Third, there may be errors in recall when investigating AR through a questionnaire, without physical examination, and there may be differences in the tolerance reported by children for specific symptoms. In order to overcome these limitations, it is necessary to investigate serum eosinophil percentage and total IgE concentration in a longitudinal study; these parameters have previously been associated with the development of allergic symptoms and allergic sensitization [25].

## Conclusion

The presence of rhinological manifestations declines with age in children sensitized to allergens. Longitudinal studies are warranted to clarify the clinical significance of asymptomatic sensitization.

## Supporting information

S1 Table Questionnaire for rhinitis symptoms from the Korean International Study of Asthma and Allergies in Childhood questionnaire. (DOCX)
