# Supplementary material for: Chronological changes in rhinitis symptoms present in school-aged children with allergic sensitization
Source: PLoS One. 2019 Jan 17;14(1):e0210840. doi: 10.1371/journal.pone.0210840 (PMC6336313; doi:10.1371/journal.pone.0210840)
Supplement: S1 Table — (DOCX) [file pone.0210840.s001.docx]

**Supporting information**

S1 Table

| Questionnaire for rhinitis symptoms from the Korean International Study of Asthma and Allergies in Childhood questionnaire | |
| --- | --- |
| Korean | 지난 12개월 동안 감기나 독감을 앓고 있지 않은데도 재채기 또는 콧물 또는 코막힘 증상을 보인적이 있습니까? |
| English | “Has your child ever had a problem with sneezing, a runny nose, or a blocked nose when he/she did not have a cold or the flu?” |
